# No evidence for a relationship between breed cooperativeness and inequity aversion in dogs

**DOI:** 10.1371/journal.pone.0233067

**Published:** 2020-06-17

**Authors:** Jim McGetrick, Désirée Brucks, Sarah Marshall-Pescini, Friederike Range

**Affiliations:** 1 Domestication Lab, Konrad Lorenz Institute of Ethology, University of Veterinary Medicine, Vienna, Austria; 2 Comparative Cognition Unit, Messerli Research Institute, University of Veterinary Medicine, Medical University of Vienna & University of Vienna, Vienna, Austria; 3 Institute for Agricultural Sciences, ETH Zürich, Zürich, Switzerland; University of Jyväskylä, FINLAND

## Abstract

Inequity aversion, the resistance to inequitable outcomes, has been demonstrated in a wide variety of animal species. Inequity aversion was hypothesised to have co-evolved with cooperation but only limited evidence supports this. Dogs provide a suitable model species to test this hypothesis as dogs were previously shown to be inequity averse and dog breeds vary in the extent to which they were selected for cooperativeness. Here, we compared the response of 12 individuals of “cooperative worker” breeds with that of 12 individuals of “independent worker” breeds in the “paw task” previously used to demonstrate inequity aversion in dogs. We also compared the two breed groups’ subsequent social behaviours in a food tolerance test and free interaction session. Although subjects in both breed groups were inequity averse, we found no considerable difference between the groups in the extent of the negative response to inequity or in the impact of the inequity on subsequent social behaviours. However, we found differences between the breed groups in the response to reward omission with cooperative breeds tending to work for longer than independent breeds. Additionally, in the free interaction session, individuals of cooperative breeds spent more time in proximity to their partner in the baseline condition than individuals of independent breeds. Overall, our results do not provide support for the hypothesis that inequity aversion and cooperation co-evolved. However, they illuminate potential differences in selection pressures experienced by cooperative worker and independent worker dog breeds throughout their evolutionary history.

## Introduction

Many non-human animal species compare payoffs with social partners and display negative responses to inequity (see Brosnan [1], Brosnan and de Waal [2], McGetrick and Range [3], and Oberliessen and Kalenscher [4] for review). Such inequity aversion is thought to counteract exploitation and, consequently, to contribute to the maintenance of cooperation [5–7], particularly among non-kin. To explain the presence of inequity aversion across multiple animal species, Brosnan [6] proposed that cooperation and inequity aversion co-evolved. However, this hypothesis has not yet been conclusively verified.

Cooperation has been defined by Brosnan and de Waal [8] as the voluntary acting together of two or more individuals that brings about, or could potentially bring about, an end situation that benefits one, both, or all of them in a way that could not have been brought about individually. There is currently some, albeit limited, experimental evidence to support the hypothesis that inequity aversion and cooperation co-evolved. For example, numerous species, including chimpanzees (*Pan troglodytes*) [9,10], wolves (*Canis lupus*) [11], capuchin monkeys (*Cebus apella*) [12,13], and long-tailed macaques (*Macaca fascicularis*) [14], have exhibited inequity aversion in experimental studies and are known to cooperate naturally with conspecifics in contexts such as hunting (chimpanzees [15–18]; wolves [19,20]), food sharing (chimpanzees [17,18,21]; wolves [20,22,23]; capuchin monkeys [24]), grooming (chimpanzees [25–28]; long-tailed macaques [29,30]; capuchin monkeys [31–33]), coalitionary support (chimpanzees [34,35]; wolves [36]; long-tailed macaques [37,38]; capuchin monkeys [32]) and territorial/group defense (chimpanzees [39]; wolves [36,40,41]), even with unrelated conspecifics in some cases (e.g. chimpanzees [42–44] and possibly wolves [45]). In contrast, orangutans (*Pongo pygmaeus*) [46,47], squirrel monkeys (*Sairmiri spp*) [47], and kea (*Nestor notabilis*) [48], species that are either solitary or that (as has been argued [6,48]) do not cooperate or cooperate to a lesser degree, have not been shown to respond negatively to inequity.

However, there is also evidence that is inconsistent with this hypothesis. For example, these non-inequity averse species have been shown to cooperate in some contexts [49–56]. In fact, kea were shown to successfully perform a loose-string cooperation task and, strikingly, a trend was observed whereby inequity on a particular trial reduced their likelihood of attempting to cooperate on the next trial [52]. Thus, despite being an apparently non-inequity averse species according to the standard exchange task [48], the loose-string cooperation task with kea provides one of the few examples in the literature of inequity influencing cooperation. Such results may indicate that the context in which the inequity is tested determines the likelihood of it being observed. In a similar vein, gorillas (*Gorilla spp*) did not express the typical primate negative response to inequity in an experimental setting involving food [57], though evidence suggests that they perceive and respond to inequity during play bouts [58]. Additionally, in some species that apparently cooperate regularly with conspecifics, either there is simply weak or no evidence for the presence of inequity aversion or there are inconsistent findings [46,59–64]. Therefore, further evidence is needed to support the hypothesis that inequity aversion and cooperation co-evolved.

Domestic dogs represent a useful model species to further explore the link between inequity aversion and cooperation. Domestic dogs were the first non-primate species found to exhibit inequity aversion and are now one of the best studied species in this field (see McGetrick and Range [3] for review). Range *et al*. [65] reported that when subjects were asked to give their paw in an inequity task (the “paw task”), they stopped complying sooner, and hesitated longer, if their partner received a reward and they themselves did not, compared with the equity condition in which both dogs received an equal reward for the same task. Additionally, subjects gave up sooner in the inequity condition than in a “no-reward” control condition in which they were unrewarded for giving the paw in the absence of a partner. Unlike capuchin monkeys [13] and chimpanzees [9], however, dogs did not refuse to comply when their partner received a better quality reward of sausage compared with their own lower quality reward of bread for performing the same task. Therefore, dogs seem to possess a basic form of inequity aversion, a finding that has since been replicated in at least four more studies using the same or similar tasks [11,66–68] (but see Horowitz [69] and Brucks *et al*. [70]).

Importantly, recent studies with dogs support a link between inequity aversion and cooperation. For example, in Brucks *et al*. [66], dogs were less likely to share food with their conspecific partner in a food tolerance test immediately after experiencing inequity in the paw task. Moreover, in a free interaction session they spent less time in proximity to their partner and took longer to approach the human experimenter immediately after the experience of inequity compared with after the equity condition [66] (see also Essler *et al*. [11] for similar findings in a free interaction session). Such social consequences of inequity may reveal the pathway through which inequity influences cooperation: avoidance of former partners could be an important behaviour that allows disadvantaged subjects to terminate detrimental relationships and to find new and more favourable cooperative partnerships.

Crucially, however, dog breeds differ in the inclination to cooperate, at least with humans, having been selected for different roles [71–73] including hunting, herding, sled-pulling, and livestock guarding [74,75]. Not surprisingly, dog breeds differ in a variety of behavioural and cognitive measures [76] which may reflect the roles for which they were selected and the degree of close cooperation required for those roles. For example, Passalacqua *et al*. [77] found that the hunting and herding breeds spent more time gazing at the human in an unsolvable task than primitive and molossoid breeds [78]. Jakovcevic *et al*. [79] reported longer durations of gazing at the human face for retrievers compared with German shepherds and poodles. Wilsson and Sundgren [80] found that Labrador retrievers are more cooperative (i.e. they tend to be influenced by a human handler even without direct commands [based on Wilsson and Sundgren’s definition [80]]) than German shepherds. According to their working styles, dog breeds have been categorized into “cooperative worker” and “independent worker” breeds by Gácsi *et al*. [81] who argued that cooperative worker breeds were “selected to work in close cooperation and visual contact with human partners” (e.g. herding dogs and gundogs), while independent worker breeds were “selected to work while visually separated from human partners” (e.g. hounds, livestock guarding dogs, and sled dogs). Moreover, they found that cooperative workers perform better at following a human point cue than independent workers and mongrels. Given this variation in cooperativeness, dogs offer a unique opportunity to test the hypothesis of the co-evolution of inequity aversion and cooperation.

Using dog breeds to test the hypothesised link between inequity aversion and cooperation offers an additional advantage over testing such hypotheses across different species. Particular experimental tasks might not be perceived in the same way by different species, and similar behavioural responses from different species might not result from the same underlying mechanisms [82]. A comparative study of dog breeds which vary in cooperativeness overcomes these issues to some degree, as dogs represent a single species across which a particular task should be perceived similarly and across which the same behaviour is more likely to represent the same underlying mechanisms.

Based on the hypothesis that inequity aversion and cooperation co-evolved, one may predict a stronger response to inequity in those dog breeds selected for greater cooperativeness. The results of one study already match this prediction. In a revised test of inequity aversion in which a subject had to choose between a fair trainer who distributed rewards equally between itself (the subject) and a partner dog, and an unfair trainer who distributed rewards unequally, Horowitz [69] found that cooperative worker breeds were more likely than independent worker breeds to choose the fair trainer. Although this result provides evidence that the cooperativeness of the dog breed may be positively correlated with an aversion to inequity, this difference between the breed groups was not statistically significant. More importantly, however, it is questionable whether the experiment actually assessed inequity aversion (see Horowitz [69] and McGetrick and Range [3] for discussion). Thus, it remains to be seen whether cooperative and independent worker breeds truly differ in the extent to which they are inequity averse.

In this study, we set out to investigate whether breed group (cooperative or independent) affects the degree of inequity aversion in dogs. We used the “paw task” originally devised by Range *et al*. [65] and we categorized the breeds according to whether they are considered cooperative workers (i.e. those that work in continuous visual contact with humans) or independent workers (i.e. those that do not work in continuous visual contact with humans), as per Gácsi *et al*. [81].

It is possible that breed differences in inequity aversion are also reflected in the social consequences of inequity. For example, if subjects of a particular breed group are more inequity averse, they may be less likely than the other breed group to co-feed with their partner after the experience of inequity. Moreover, they may be more likely to avoid their partner or the experimenter in a free interaction session. In order to detect any such social consequences, as in Brucks *et al*. [66], we included a food tolerance test and a free interaction session, in succession, immediately after each condition of the paw task.

## Methods

### Subjects

Twenty-four subjects were tested in this study. Twelve of these subjects belonged to “cooperative worker” breeds (4 Australian shepherds, 4 border collies, 2 Labrador retrievers, and 2 rough collies; 6 females and 6 males). The other twelve belonged to “independent worker” breeds and were also primarily “ancient” and “spitz” breeds (see Parker *et al*. [73] and von Holdt *et al*. [72]) (2 akita inus, 1 basenji, 4 Siberian huskies, 5 shiba inus; 8 females and 4 males). The mean age (± S.E.M.) of dogs included in this study was 5.22 ± 0.63 years. Subjects were tested with a familiar conspecific in the paw task, food tolerance test, and free interaction session (see below); for all except one “cooperative worker” subject, this was a household partner. In order to be included in the study, subjects had to be able to give their paw to the experimenter 10 times on command in return for a food reward (see below).

### Experimental setup and procedure

**Fig 1:**
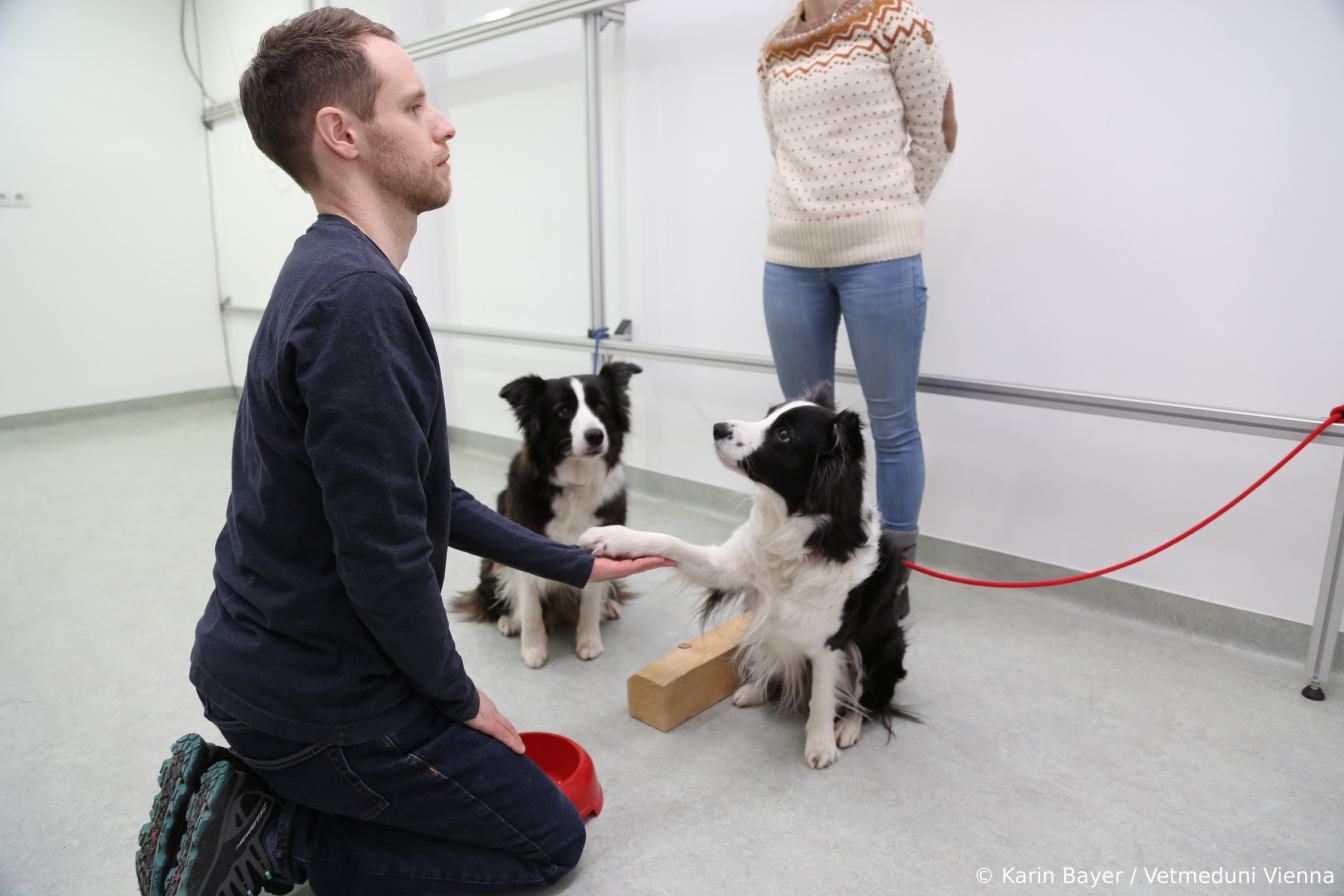
Paw task. The experimenter kneels in front of the two dogs, with a bowl of food, and asks each dog for its paw, before rewarding it, or not, depending on the condition.

This experiment was carried out between January and July 2016 in a 7 m x 6 m room in the Clever Dog Laboratory, located at the University of Veterinary Medicine, Vienna. Experiments were recorded using four cameras (with one positioned in each corner of the room). All procedures carried out here were approved by the ‘Ethik und Tierschutzkomission’ of the University of Veterinary Medicine (protocol numbers: ETK-08/07/97/2013; ETK-07/11/2016). Additionally, owners were required to sign consent forms prior to testing. The experimenter and owner in Fig 1 have both given written informed consent (as outlined in the PLOS consent form) to publish this image.

#### Paw task

The procedure carried out here follows that originally used by Range *et al*. [65] and subsequently employed by Brucks *et al*. [66] (see Fig 1). The subject and partner were positioned approximately 30 cm apart with their leashes tied to metal poles (which were approx. 2 m apart) at the wall. The leashes of the two dogs were tied at approximately the same length (approx. 1.5 m); thus, both dogs were positioned at a similar distance from the wall and although the leashes prevented them from leaving the experimental setting completely, they could move away from the experimenter. The dogs were separated by a wooden block (60 cm x 10 cm x 10 cm) placed on the ground as a marker of the midpoint; this did not prevent dogs from having physical contact with each other. The owner of the two dogs (or both owners in the case of one of the cooperative dyads) stood against the wall, in between the two dogs, and did not interfere during the experiment. The experimenter knelt on the ground facing both dogs approximately 30 cm away and avoided direct eye contact with both dogs throughout the paw task session. A red food bowl (30 cm in diameter) was positioned on the floor in front of the experimenter and contained small pieces of sausage. The experimenter asked the dogs to give their paw alternately (30 times each) with an outstretched hand and the verbal command “Pfote!”. The partner, whenever present, was always rewarded with a piece of sausage for giving the paw, while the subject was either rewarded or not depending on the condition (see Table 1). The experimenter always began by asking the partner for their paw; thus, after alternating between the partner and subject, the session always ended with the subject. If dogs were not in a sitting position before being asked to give their paw, they were requested to sit with an outstretched finger and the verbal command “Sitz!”. If subjects refused to sit, the command was repeated a further 9 times with the subject’s name being called after the 5^th^ command. The session was terminated after 10 disobeyed sit commands. However, if the subject changed position considerably in the middle of this sequence of sit commands, the experimenter started again with 10 new sit commands. If subjects refused to give their paw once seated, the paw command was repeated a further 10 times, with the subject’s name being called after the 5^th^ repetition. The session was terminated following refusal by the subject to give their paw on these commands. A second experimenter sat on a chair approximately 2 m away and marked a score sheet each time the subject and partner gave their paw, and each time a paw or sit command was given. One session was carried out per day and consisted of a maximum of 60 trials (i.e. 60 obeyed paw commands; one condition). On one occasion, for one pair of Siberian huskies, two conditions were conducted on the same test day.

**Table 1:** Conditions used in the paw task.

Condition	Rewarded?^a^	
	Subject	Partner
***Social***		
**Equity (ET)**	+	+
**Reward Inequity (RI)**	-	+
***Asocial***		
**No-reward (NR)**	-	Not present^b^

^a^Rewards used were pieces of sausage.

^b^A piece of sausage was moved to the partner’s empty position after each trial in which the subject gave the paw.

To be considered suitable for participation in this study, the subject and partner were required to give their paw to the experimenter, alternately, ten times each for a piece of sausage each time. This was carried out on the dogs’ first visit to the lab. If the dogs were not successful, they were asked a subsequent ten times each, or the owner was asked to continue training the dog at home and they were retested on a second visit. An assessment was not carried out with one of the huskies beforehand; however, the owner was familiar with the experimental procedure and had worked with this individual beforehand. One subject (an akita inu) was tested in the paw task while lying down as it was not comfortable sitting according to the owner; the sit criteria was only applied to the partner (another akita inu) in this case. The first session for this dyad was repeated when they had completed all other sessions, as the sit criteria for this subject had been applied to both dogs in the first session.

Dogs were tested using the equity (ET), reward inequity (RI), and no-reward (NR) conditions previously used by Range *et al*. [65] (see Table 1). In the ET condition, the subject and the partner received a reward of the same value each time they gave the paw. In the RI condition the partner received a reward each time it gave the paw, whereas the subject received nothing for giving the paw. In the NR condition, the subject, again, received no reward for giving the paw but no partner was present. Although no partner was present in the NR condition, a piece of food was lifted from the bowl and moved towards this empty position after each trial completed by the subject, as though feeding the partner; this piece of food was then moved back to the bowl. This movement was included, as in the previous studies [65,66], to control for the movement of the food that occurs in the RI condition. Before handing out a food reward, the reward was briefly held in between the two dogs for both to see. Each condition was carried out on a separate day and lasted for 60 trials (30 trials per dog) unless the subject refused to give their paw or to sit before all trials were complete. The only reward type used in this study was sausage except for one pair in which both individuals were rewarded with dry food, on the owner’s request.

Unlike in the study of Range *et al*. [65], here, the ET condition was repeated a second time so as to avoid subjects experiencing more unrewarded than rewarded conditions. An ET condition was carried out on the first test day followed on subsequent test days by either the RI or NR condition, the second ET condition, and then again by either the RI and NR condition depending on which of these they had already experienced. Five subjects in each of the two breed groups experienced the RI condition before the NR condition while seven subjects in each breed group experienced the NR condition before the RI condition. In most cases, both dogs in the dyad were considered subjects in the study as they were both breeds of interest. In these cases, dogs in a dyad were treated as subjects in concurrent or successive sessions depending on the condition. For example, the results of the two ET conditions were counted for both dogs and the roles (i.e. which dog was asked for their paw first) were reversed for the second ET condition. However, RI conditions were carried out in successive sessions (as the two dogs are treated differently in the RI condition and the response to inequity can only be assessed in one individual at a time).

Throughout the study, in ET sessions for dyads in which both individuals were considered subjects, if one of the individuals in the pair stopped giving their paw after the set number of paw or sit commands, the experimenter continued asking the other individual for the paw until the 30 trials were complete, or until it too gave up. This situation arose in seven ET sessions; in two of these, the subject and partner gave up at the same point. Additionally, one of the shiba inus experienced two ET sessions and the NR condition before being placed with a new experimental partner due to the unreliable performance of the original partner. Consequently, its NR condition was carried out before the two ET conditions with the new experimental partner.

#### Food tolerance test

Immediately after termination of the ET and RI conditions, and removal of the food bowl from the room, a second bowl (20 cm diameter) containing approximately 20 slices from a single sausage was shown to the dogs before being placed on the ground, equidistant from both dogs (approximately 2 m away). The dogs were released at the same time by the two experimenters and were allowed to approach the bowl and feed together. The owner(s) remained in the same position as during the paw task. After the sausage had been eaten, once both dogs were 1 m away, the bowl was removed by one of the experimenters. Two dogs (i.e. one dyad) did not participate in this test in order to avoid potentially aggressive interactions. In two ET and two RI conditions, the experimenter began moving towards the bowl before both dogs had moved 1 m away; in these cases, both dogs had finished eating (e.g. they were standing beside the bowl or facing or moving away) and the session was considered finished the moment the experimenter’s hand touched the bowl.

#### Free interaction session

Following the food tolerance test, the second experimenter left the room. The primary experimenter and the dog owner(s) knelt or sat on the ground, approximately 2.5 m apart, facing each other, for 5 minutes. The session began when the experimenter and owner’s knees had touched the floor and the door of the room was closed (in some cases the owner hunkered or sat; thus, the session began when the owner settled in a hunkered or seated position and the experimenter’s knees touched the floor). Both dogs were already free in the room and were allowed to roam freely around the room during this period. The experimenter and owner(s) were permitted to pet the dogs if they approached but were not permitted to actively attract the dogs’ attention or to call the dogs over.

### Behaviour coding

Tasks were coded using Solomon Coder beta 17.03.22 (copyright 2017 by András Péter; https://solomoncoder.com/). In the paw task we coded the number of times the subjects gave the paw on command, the number of times they were given a paw command, the number of times they were given a sit command, and the number of stress behaviours they exhibited. The stress behaviours we counted included lip-licking, yawning, scratching, and stretching. All stress behaviours exhibited by the subject within each session were counted; however, lip-licking which occurred within a 2 second period immediately after the receipt of food was excluded. We coded the duration of time spent co-feeding (defined as both dogs having their snouts in the bowl at the same time) in the food tolerance test. For the free interaction sessions, based on measures which revealed significance in the study of Brucks *et al*. [66], we coded the duration of proximity (less than one body length) to the conspecific partner and the latency to have contact with the experimenter (latency was measured as the time taken from the beginning of the free interaction session until the subject had contact with the experimenter).

### Statistical analysis

Statistical analyses were carried out using R (version 3.6.1[83]). The package “lme4” [84] was used for statistical analyses and the package “ggplot2” [85] was used for creation of plots, unless otherwise stated.

A generalised linear mixed model (GLMM) with a binomial error distribution was employed to analyse the number of completed trials. The response variable comprised the number of planned trials which were completed versus the number of planned trials which were not completed. This response was entered in the model using the “cbind” function. Condition (ET, RI, or NR), breed group (cooperative or independent), age, and sex were included as fixed effects in the model with an interaction between condition and each of the remaining three variables (see Table 2). Random effects of observation ID (i.e. the identity of each individual data point), and subject ID nested within dyad ID (i.e. it was indicated that each subject belonged within a particular dyad), were included in the model.

**Table 2:** Summary of response variable, fixed effects, random effects, offset term, and frailty term included in each model.

**Paw Task**			
*Response variable*	*Fixed effects*^*a*^	*Random effects*^*b*^	*Offset term*
cbind(no. of planned trials completed, no. of planned trials not completed)	Condition*Breed group;	Observation ID;	-
	Condition*Age;	Dyad ID/Subject ID	
	Condition*Sex		
No. of paw + sit commands	Condition*Breed group;	Dyad ID/Subject ID	Log(no. of trials completed)
	Condition*Age;		
	Condition*Sex		
No. of stress behaviours	Condition*Breed group;	Dyad ID/Subject ID	Log(test duration)
	Condition*Age;		
	Condition*Sex		
**Food Tolerance Test**			
*Response variable*	*Fixed effects*	*Random effects*	*-*
Duration of co-feeding	Condition*Breed group;	Dyad ID/Subject ID	-
	Condition*Age;		
	Condition*Sex		
**Free Interaction Session**			
*Response variable*	*Fixed effects*	*Random effects*	*Frailty term*
Duration of proximity to the partner	Condition*Breed group;	Dyad ID/Subject ID	-
	Condition*Age;		
	Condition*Sex		
Latency to have contact with experimenter	Condition*Breed group;	-	Subject ID
	Condition*Age;		
	Condition*Sex		

^a^An asterisk between two fixed effects indicates the inclusion of both fixed effects, and an interaction between them in the model.

^b^A solidus indicates that one effect is nested in the other in the model; in most of these models subject ID was nested within dyad ID.

The number of paw and sit commands (combined) given by the experimenter and the number of stress behaviours displayed by the subjects were analysed using negative binomial GLMMs due to overdispersion in models with a poisson error distribution (we tested for overdispersion using a function kindly provided by Roger Mundry). The same fixed effects as above were included in the models. Subject ID nested within dyad ID was included as a random effect in both models. For analysis of the number of commands, an offset term of the log of the number of trials completed was included. For analysis of the number of stress behaviours displayed, an offset term of the log of the test duration was included. Thus, the number of commands and the number of stress behaviours were corrected for the number of trials completed and the test duration respectively.

The duration subjects spent feeding with their partner (co-feeding) was analysed using a generalised additive model for location, scale, and shape (GAMLSS) using the R package “gamlss” [86] and specifying a zero-adjusted gamma distribution (“ZAGA”). The same fixed and random effects as in the previous analyses were applied (with the exclusion of the observation level random effect). Diagnostics were carried out by visual inspection of a qqplot, a residual vs index plot, and a worm plot.

Given that some dyads consisted of two subjects, both being observed, the duration of co-feeding was duplicated in the ET condition (i.e. it was the same value for both dogs in the dyad). To avoid duplicating data in the analysis, we ran five models in which duplicated observations were semi-randomly removed such that both dogs in a dyad retained an observation for the ET condition but it was different to that of their partner’s (i.e. if subject 1 was assigned observation 1, subject 2 from the same dyad was assigned observation 2 etc.). The mean output from five full-null model comparisons is reported (see below for description of the full-null model comparison).

Duration of proximity to the conspecific partner was analysed using a linear mixed effects model (LMEM). The fixed and random effects were identical to those in the models used to analyse the food tolerance test. The same issue of duplicated data points in the ET condition, mentioned above for duration of co-feeding, applied here; therefore, we carried out the same procedure of running five models in which duplicated observations were semi-randomly removed, and we subsequently took the mean output from five full-null model comparisons. A boxcox transformation method was applied to all five models using the package “MASS” [87]. Visual inspection of diagnostic plots including histograms, qqplots, and residual vs fitted plots, was used to assess normality and homoscedasticity, in combination with a Shapiro-Wilk normality test using the R function “shapiro.test”, and the Breusch-Pagan test in the package “lmtest” [88] to asses homoscedasticity.

Latency to contact the experimenter in the interaction session was assessed with a Cox proportional hazards regression model [89] using the “survival” [90,91] and “survminer” [92] packages. The same fixed effects as above were applied here and a frailty term of subject was included to account for repeated measures. The assumption of proportional hazards was assessed using the “cox.zph” function and Schoenfeld residual plots in the “survival” [90,91] package. The cumulative incidence plot (Fig 5) was created using the “ggsurvplot” function in the packages “survminer” [92] and “ggplot2” [85]. Table 2 comprises a summary of the terms included in each model.

Interpretation of model output to assess the effect of breed group, or the interaction between condition and breed group, was conditional on obtaining a significant difference between a given full model and a respective null model. A full-null model comparison was conducted to avoid cryptic multiple testing and was based on a likelihood ratio test (using the R function “anova” and setting the “test” argument to “Chisq”; in the case of GAMLSS models full-null model comparisons were conducted using the function “lrtest” in the package “lmtest” [88]). The full model in all cases contained the terms of interest (i.e. breed group and its interaction with condition). Breed group and its interaction with condition was omitted from the null model. In the absence of a significant interaction between breed group and condition, we also removed all interactions from the full model to determine the overall effect of condition to investigate whether our results matched those from previous paw task studies (e.g. Range *et al*. [65]; Brucks *et al*. [66]). Significant results from all analyses remained significant after sequential Bonferroni correction for multiple testing, unless stated otherwise.

A second experimenter coded 20% of the videos and inter-observer reliability was assessed with the intra-class correlation coefficient using the package “irr” [93] (Intra-class correlation coefficient [ICC, consistency]: paw task: number of times the paw was given on command: ICC (two-way, consistency) = 1, p < 0.001; number of paw commands: ICC (two-way, consistency) = 0.999, p < 0.001; number of sit commands: ICC (two-way, consistency) = 0.999, p < 0.001; stress: ICC (two-way, consistency) = 0.676, p = 0.001; food tolerance test: duration of co-feeding: ICC (two-way, consistency) = 0.998, p < 0.001; interaction session: duration of proximity to conspecific partner: ICC (two-way, consistency) = 0.964, p < 0.001; latency to have contact with the experimenter: ICC (two-way, consistency) = 0.684, p < 0.001).

## Results

### Paw task

**Fig 2:**
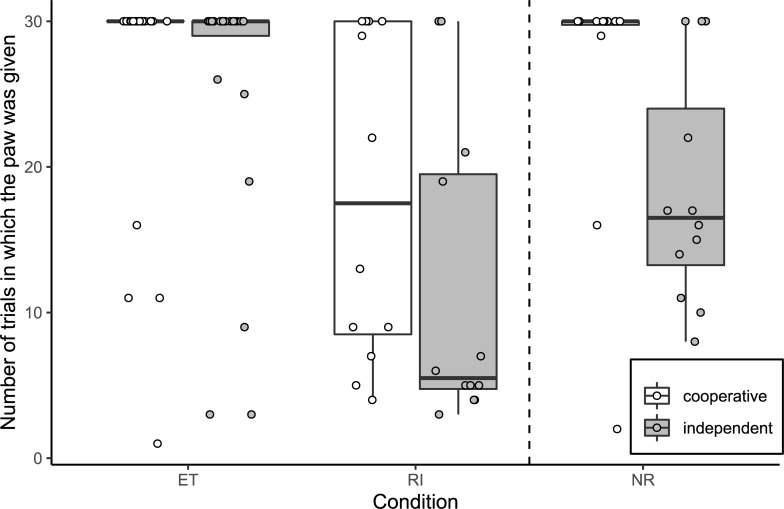
Number of times the paw was given on command by cooperative worker (N = 12) and independent worker (N = 12) breeds in each condition of the paw task. Boxes display the interquartile range, black horizontal bars represent the median, whiskers represent the range of data points within 1.5 times the interquartile range from the upper and lower hinge, and circles represent individual data points; dashed, vertical line separates social and asocial conditions. ET, equity; RI, reward inequity; NR, no-reward.

Comparison of the full and null model analysing the number of times subjects gave the paw in the paw task revealed a trend (likelihood ratio test: χ^2^ = 6.77, df = 3, p = 0.080; see Fig 2). The model output indicated no significant interaction between condition and breed group (with “RI” as the reference level for condition, and “cooperative” as the reference level for breed group: GLMM: ET:independent: β = 0.84, S.E. = 1.81, p = 0.644; NR:independent: β = -2.30, S.E. = 1.94, p = 0.236). We therefore removed the interaction between breed group and condition from the model. There was a significant effect of breed group on the response (GLMM: cooperative vs independent: β = -3.56, S.E. = 1.71, p = 0.037; not significant after correction for multiple testing [adjusted alpha = 0.017]). We subsequently removed all interactions from the full model to investigate whether condition had a significant effect; the number of times the subjects gave the paw in the RI condition was significantly lower than that in the ET and NR conditions, as per previous paw task studies [65,66,68] (GLMM: RI vs ET: β = 5.55, S.E. = 0.96, p < 0.001; RI vs NR: β = 2.80, S.E. = 0.93, p = 0.003).

The full-null model comparison revealed no significant effect of group on the number of commands issued per trial (likelihood ratio test: χ^2^ = 2.69, df = 3, p = 0.442; see S1 Fig). We subsequently removed all interactions from the full model and confirmed an effect of condition: more commands were required in the RI condition than both the ET and NR conditions (GLMM: RI vs ET: β = -0.55, S.E. = 0.09, p < 0.001; RI vs NR: β = -0.40, S.E. = 0.10, p < 0.001).

In the case of the number of stress behaviours displayed, the full-null model comparison obtained a non-significant trend for the effect of group (likelihood ratio test: χ^2^ = 6.77, df = 3, p = 0.080; see S2 Fig). The model output revealed no significant interaction between condition and breed group (with “RI” as the reference level for condition, and “cooperative” as the reference level for breed group: GLMM: ET:independent: β = -0.60, S.E. = 0.46, p = 0.196; NR:independent: β = 0.37, S.E. = 0.56, p = 0.511). We removed the interaction between condition and breed group from the model but no significant effect of breed group was present (GLMM: cooperative vs independent: β = 0.31, S.E. = 0.22, p = 0.148) (singular fit issues were reported for this model and, in such cases, likelihood ratio tests may not be appropriate; the null-full model comparison result and the model estimates may be unreliable [84]). Visual inspection of the data in a boxplot (S2 Fig) indicates a pattern in keeping with that for the number of times the paw was given: independent breeds appear to exhibit slightly more stress behaviours than cooperative breeds, specifically in the RI and NR conditions.

### Food tolerance test

**Fig 3:**
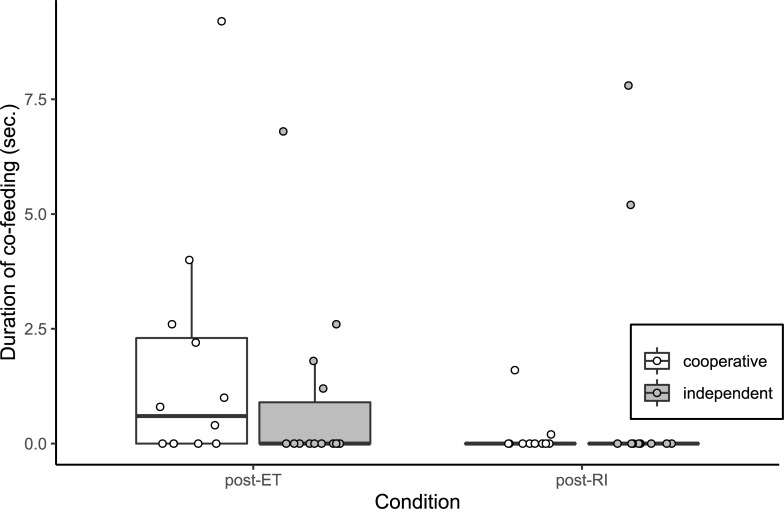
Duration of co-feeding between subject and partner in the food tolerance test for cooperative worker (N = 10) and independent worker (N = 12) breeds after two conditions of the paw task. Boxes display the interquartile range, black horizontal bars represent the median, whiskers represent the range of data points within 1.5 times the interquartile range from the upper and lower hinge, and circles represent individual data points. ET, equity; RI, reward inequity.

The mean result from five full-null model comparisons for the duration of co-feeding in the food tolerance test was significant (likelihood ratio test: χ^2^ = 8.31, df = 2, p = 0.016; see Fig 3). Therefore, we assessed the model output from one randomly selected model. There was a significant interaction between condition and breed group (with “ET” as the reference level for condition, and “cooperative” as the reference level for breed group: GAMLSS: β = 2.90, S.E. = 0.87, p = 0.002); the duration of co-feeding was significantly lower after the RI condition than after the ET condition for cooperative breeds but not for independent breeds (GAMLSS: ET vs RI: cooperative: β = -3.73, S.E. = 1.21, p = 0.004; independent: β = -0.84, S.E. = 1.15, p = 0.469). The interpretability of this result is, however, limited by the low levels of co-feeding observed (e.g. the median duration of co-feeding for independent breeds was 0 seconds). Additionally, there was no significant difference between the two breed groups in the ET condition (GAMLSS: β = 0.01, S.E. = 0.46, p = 0.980).

### Free interaction session

**Fig 4:**
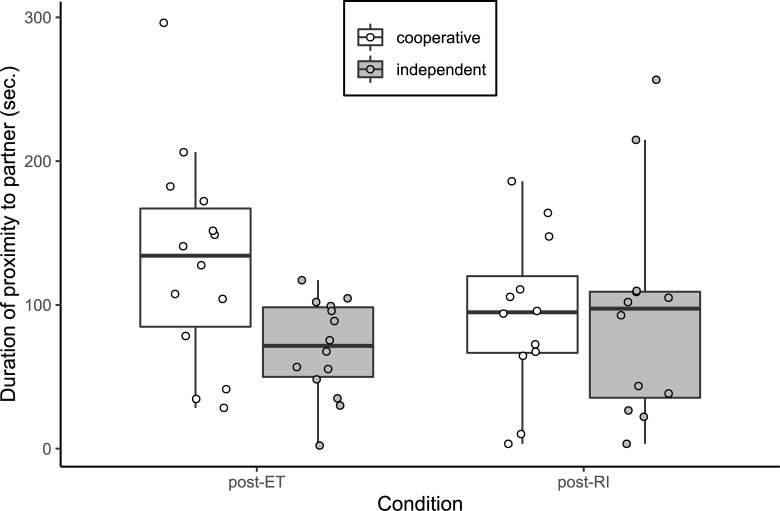
Duration spent in proximity to the conspecific partner (sec.) in the interaction session after the two social conditions of the paw task, for cooperative worker (N = 12) and independent worker (N = 12) breeds. Boxes display the interquartile range, black horizontal bars represent the median, whiskers represent the range of data points within 1.5 times the interquartile range from the upper and lower hinge, and circles represent individual data points. ET, equity; RI, reward inequity.

For analysis of the duration spent in proximity to the partner, the mean full-null model comparison revealed a significant effect of breed group or its interaction with condition (likelihood ratio test: χ^2^ = 7.29, df = 2, p = 0.034; see Fig 4). We assessed the model output from all five models and for each result we present the average of the output from the five models. The model output indicated a trend for the interaction between condition and breed group (LMEM: β = 40.15, S.E. = 19.81, p = 0.050). Duration of proximity to the partner was lower after the RI condition than after the ET condition for cooperative breeds, whereas for independent breeds this duration was higher after the RI than after the ET condition, though neither of these results were significant (LMEM: cooperative: β = -17.45, S.E. = 22.45, p = 0.448; independent: β = 23.30, S.E. = 23.79, p = 0.341). Cooperative breeds spent significantly more time than independent breeds in proximity to their partner after the ET condition (LMEM: β = -38.07, S.E. = 13.77, p = 0.011).

**Fig 5:**
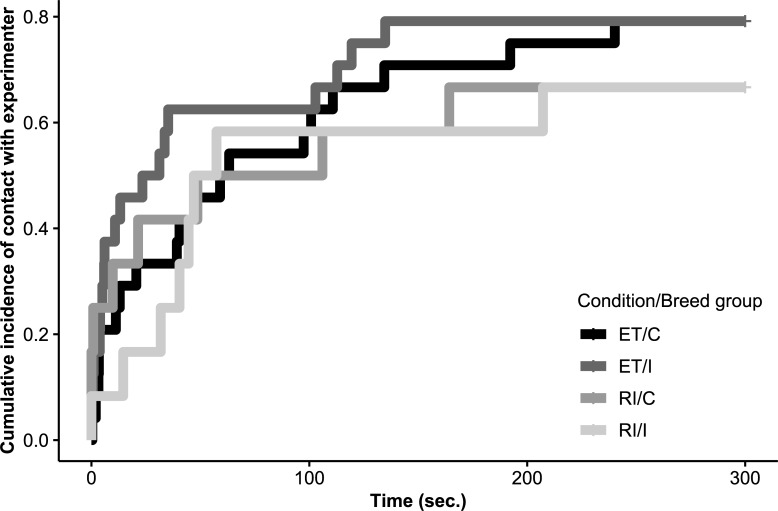
Cumulative incidence of first contact with the experimenter (as proportion of observations) for subjects of cooperative worker (N = 12) and independent worker (N = 12) breeds in the interaction session after both social conditions of the paw task. Derived from Kaplan-Meier estimates. ET, equity; RI, reward inequity; C, cooperative worker breeds; I, independent worker breeds.

A comparison of a null and a full Cox proportional hazards model indicated no significant effect of group on the latency to have contact with the experimenter (likelihood ratio test: χ^2^ = 0.34, df = 2, p = 0.844; see Fig 5). We subsequently assessed the output of the full model without interactions to determine whether condition had an effect on the latency to have contact with the experimenter (the factor “sex” was removed from the model due to violation of model assumptions). There was no significant effect of condition (Cox proportional hazards model: β = -0.50, S.E. = 0.33, p = 0.12). Results are summarised in Table 3.

**Table 3:** Summary of results.

Variable	Interactions	Breed group	Condition
***Paw Task***			
Paw giving	-	C > I	ET, NR > RI
Commands (paw + sit)	-	-	RI > ET, NR
Stress	-	I > C*	-
***Food tolerance test***			
Co-feeding	C: ET > RI	-	-
	I: ET = RI		
***Free interaction session***			
Duration of proximity to partner	C (ET) > I (ET)	-	-
Latency to have contact with experimenter	-	-	-

The direction of significant results is indicated. Non-significant results are omitted. ET, equity; RI, reward inequity; C, cooperative; I, independent.

*Significant effect of group or its interaction with condition indicated by the likelihood ratio test but not model output. Visual inspection of boxplot suggests a greater number of stress behaviours in independent breeds than in cooperative breeds.

## Discussion

Overall, subjects in the current study were inequity averse, as per previous paw task studies [65,66,68]; however, we obtained no convincing evidence for breed differences in the extent of the negative response to inequity, or in the effect of inequity on subsequent social behaviours. Thus, our results do not lend support to the hypothesised link between inequity aversion and cooperation [6].

Although we have not provided evidence here for a relationship between inequity aversion and cooperation, it is worth highlighting that several explanations for the evolution of cooperation exist [94–96] and inequity aversion is unlikely to be involved in all recognised forms of cooperation [7]. For example, Brosnan and Bshary [7] recently suggested that inequity aversion could influence forms of cooperation such as direct reciprocity [95] (i.e. helping those that help you), negative pseudo-reciprocity [97] (i.e. cooperating to avoid the self-serving action of a partner), and indirect forms of cooperation [98] (i.e. those based on reputation effects), but should not play a role in by-product mutualism [96,99,100] (i.e. helping another individual as a by-product of a self-serving action) and direct positive pseudo-reciprocity [97,101] (i.e. helping another individual due to return benefits produced by that individual as a by-product of a self-serving act). Dog-human cooperation may correspond taxonomically with the latter forms of cooperation. Thus, a lack of evidence for cooperative breeds being more inequity averse than independent breeds does not necessarily refute the hypothesised co-evolution of inequity aversion and cooperation.

Additionally, it should be pointed out that the extent to which firm conclusions can be drawn from the paw task regarding breed differences in inequity aversion is limited due to ceiling effects. In the current study, the maximum number of trials a subject could complete was 30 and many of the subjects in the cooperative group completed all trials in the ET and NR conditions. The degree of a negative response to inequity cannot be determined accurately if subjects complete all trials in each condition, or if they complete all trials in the ET and NR conditions (it is noteworthy that a similar issue exists in numerous studies that fail to find inequity aversion [see, for example, Range *et al*. [65], McAuliffe *et al*. [63], Heaney *et al*. [48], Krasheninnikova *et al*. [102]]). It is still possible that individuals in the cooperative group react more strongly in the RI condition relative to the NR condition than do independent breeds, making them (cooperative breeds) more inequity averse. Continuation of each condition until subjects give up, would allow us to accurately compare the performance across conditions and to determine the extent of the negative response to inequity. However, there was also no significant effect of an interaction between condition and breed group on the number of commands issued or the number of stress behaviours displayed in the paw task, further suggesting the absence of a difference between breed groups.

Regarding the free interaction session, the lack of an effect of inequity is surprising as it is in conflict with the findings of Brucks *et al*. [66]. One clear difference between our study and that of Brucks *et al*. [66] is that we provided both the subject and partner with a handful of sausage immediately after each condition of the paw task (i.e. immediately before the food tolerance test and free interaction session), whereas Brucks *et al*. [66] did not. It is conceivable that the provision of a handful of food in our study diminished subjects’ potentially negative attitude towards the partner or experimenter, thereby decreasing the likelihood of social consequences of the inequity emerging. Although, it is unclear to what extent giving food at the end of the paw task can influence subsequent social behaviours, as our results in the food tolerance test, at least for cooperative breeds, do match those of Brucks *et al*. [66].

Importantly, our conclusions in this study rest on the premise that the two breed groups differ in cooperativeness. However, cooperative worker and independent worker breeds may not actually differ in cooperativeness. Comparison of cooperative and independent dog breeds has become popular in recent years [69,81,103] but the nature of the behavioural difference that identifies one group as being more cooperative and the other group less cooperative is not clear. It is also important to highlight that cooperative and independent worker breeds were all selected to work for or with humans; thus, they could all be considered cooperative, with variation in the context in which the cooperation occurs rather than the degree of cooperativeness. In fact, this categorization system has already elicited criticism (see, for example Udell *et al*. [104]), particularly due to its subjectivity. The fact that differences have been found between these groups in behavioural or cognitive tasks [81,103] suggests that the distinction is meaningful in some sense but further elucidation of the differences between these breeds, and how they relate to cooperativeness, is required.

Our results provide some insight into potential differences between the two breed categories. For example, in the paw task, subjects of the independent group gave the paw fewer times than subjects of cooperative breeds. This difference is particularly evident in the unrewarded conditions and could indicate breed differences in extinction (i.e. a process whereby performance of a behaviour decreases if it is no longer reinforced [105]). Jakovcevic *et al*. [79], in fact, found differences between breeds in extinction of gazing to the human face, with retrievers gazing more at the human face during an extinction phase than German shepherds and poodles. Although these breed differences are not entirely in line with the cooperative-independent divide, and the behaviour specifically related to gazing at the human face, they do provide some evidence for breed differences in extinction.

An alternative to the suggested difference in extinction is that the breed groups may differ in their willingness or motivation to work with a human. Selection may, for example, have increased the compulsion of cooperative workers to comply with a human’s command, or it may have increased the degree to which cooperative workers find interaction with a human rewarding.

In addition to the breed difference in the paw task, we observed a significant difference between breeds in the baseline duration of proximity to the conspecific partner in the free interaction session. Cooperative breeds spent significantly more time than independent breeds in proximity to their partner after the ET condition. This may reflect some basic difference between the breed groups in sociability, though given that the difference is not considerable, and that it was observed in a specific context, future studies should investigate this further. Moreover, given the possible indication of a difference in sociability, it could also be worthwhile to investigate breed differences in co-feeding further: although there was no significant difference between the breed groups in the baseline (ET) condition, the floor effects for duration of co-feeding limit our ability to observe any potential differences. Additionally, although strong and significant effects of inequity were not observed in the duration of proximity to the partner, the lack of a difference between the breed groups after the RI condition, despite small differences after the ET condition, could indicate that the different breed groups cope with inequity by applying different behavioural strategies. However, given non-significant results and weak effects, such interpretation is highly speculative.

In conclusion, our results do not indicate that the extent to which dogs are inequity averse differs between cooperative worker and independent worker breeds and, therefore, do not lend support to the hypothesis that inequity aversion and cooperation co-evolved [6]. However, our results provide some evidence for basic breed group differences in the tendency to work without rewards, and possibly general sociability, which could offer fruitful areas of investigation for future studies focused on understanding dog breed differences.

## Supporting information

S1 Fig Number of commands (paw and sit) issued per trial by the experimenter for cooperative worker (N = 12) and independent worker (N = 12) breeds in each condition of the paw task. Boxes display the interquartile range, black horizontal bars represent the median, whiskers represent the range of data points within 1.5 times the interquartile range from the upper and lower hinge, and circles represent individual data points; dashed, vertical line separates social and asocial conditions. ET, equity; RI, reward inequity; NR, no-reward.
(EPS)

S2 Fig Number of stress behaviours displayed (corrected for test duration) by subjects of cooperative worker (N = 12) and independent worker (N = 12) breeds in each condition of the paw task. Boxes display the interquartile range, black horizontal bars represent the median, whiskers represent the range of data points within 1.5 times the interquartile range from the upper and lower hinge, and circles represent individual data points; dashed, vertical line separates social and asocial conditions. ET, equity; RI, reward inequity; NR, no-reward.
(EPS)

S1 Dataset (XLSX)
